# *Lactobacillus reuteri* ZJ617 maintains intestinal integrity via regulating tight junction, autophagy and apoptosis in mice challenged with lipopolysaccharide

**DOI:** 10.18632/oncotarget.20536

**Published:** 2017-08-24

**Authors:** Yanjun Cui, Li Liu, Xiaoxiao Dou, Chong Wang, Wenming Zhang, Kan Gao, Jianxin Liu, Haifeng Wang

**Affiliations:** ^1^ Institute of Animal Nutrition, College of Animal Science and Technology, Zhejiang A & F University, Lin’an 311300, P.R. China; ^2^ College of Animal Science, MOE Key Laboratory of Molecular Animal Nutrition, Zhejiang University, Hangzhou 310029, P.R. China

**Keywords:** *lactobacilli*, intestinal barrier, apoptosis, autophagy, mice

## Abstract

Live probiotics are effective in reducing gut permeability and inflammation. We have previously reported that *Lactobacillus reuteri* ZJ617 (ZJ617) with high adhesive and *Lactobacillus rhamnosus* GG (LGG) can ameliorate intestine inflammation induced by lipopolysaccharide (LPS). The present study was aimed at elucidating the roles of ZJ617 and LGG in alleviating the LPS-induced barrier dysfunction of ileum in mice. Six C57BL/6 mice per group were orally inoculated with ZJ617 or LGG for one week (1× 10^8^ CFU/mouse) and intraperitoneally injected with LPS (10 mg/kg body weight) for 24 h. The results demonstrated that pretreatment with ZJ617 and LGG attenuated LPS-induced increase in intestinal permeability. The probiotics supplementation suppressed LPS-induced oxidative stress. Both ZJ617 and LGG strongly reversed the decline of occludin and claudin-3 expression induced by LPS challenge. ZJ617 relieved LPS-induced apoptosis by decreasing caspase-3 activity. Noticeably, ratio of microtubule-associated light chain 3 (LC3)-II/LC3-I and LC3 activity were elevated by LPS stimulation, whereas such increases were obviously attenuated by both of the probiotics treatment. Moreover, phosphorylated mammalian target of rapamycin (p-mTOR) was significantly inhibited by LPS, whereas complementation of ZJ617 and LGG markedly increased the expression of p-mTOR. Collectively, our results indicated that ZJ617 could protect LPS-induced intestinal barrier dysfunction via enhancing antioxidant activities and tight junction and attenuating apoptosis and autophagy via mTOR signaling pathway. These findings could serve as systematic mechanisms through which probiotics promote and maintain gut homeostasis.

## INTRODUCTION

The intestinal barrier is important as a selective barrier against endogenous and exogenous noxious antigens and pathogens [1]. Impairment of this barrier can result in the increased intestinal permeability, subsequent translocation of bacteria or/and endotoxin from gut, and even systemic inflammatory response, which is the major pathogenesis for most of intestinal diseases [2]. Multiple factors, including inflammation and oxidative stress, can compromise intestinal epithelial integrity and function [3–5]. Therefore, suppression of intestinal inflammation and oxidative stress may contribute to protecting from the intestinal disruption.

*Lactobacillus* as probiotic bacteria are normal habitants of the gastrointestinal tracts in humans and animals and exert health benefits on the host including gut homeostasis and disease prevention and/or treatment. For instance, *Lactobacillus reuteri* I5007 could attenuate diquat-induced oxidative stress and maintain the gut epithelial barrier by modulating tight junction protein expression in piglets [6, 7]. The probiotic *Lactobacillus coryniformis* and *L.* gasseri SBT2055 reduced intestinal inflammation and permeability in mice, *L.* acidophilus ameliorated dextran sodium sulphate induced colitis in mice [8]. *L.* reuteri promotes anti-inflammatory activities in mice with typhlocolitis [9]. These studies suggest that Lactobacillus contribute to attenuating intestine barrier function injury, which is associated with gut inflammation and oxidative stress. The underlying mechanisms by which the probiotics exert positive effects on gut integrity remain to be elucidated.

Several reports have shed light on the mechanisms for a variety of Lactobacillus species. Many studies have shown that *Lactobacillus reuteri* I5007 and *Lactobacillus rhamnosus* GG (LGG) can maintain epithelial barrier permeability via modulation of TJ proteins expression [7, 10]. Of interest is the finding that *L. acidophilus* can directly alter epithelial barrier function by influencing the structure of TJ, which is achieved via a MAPK pathway [11]. Beyond tight junction changes, epithelial apoptosis can also contribute to barrier dysfunction [12, 13]. Thus, another study found that bacterial proteins (p75 and p40) isolated from *L.* rhamnosus cultures effectively block the induction of apoptosis in mouse colon epithelial cells, helping to enhance epithelial barrier function [14]. Beyond Lactobacillus species, a latest finding has demonstrated that *Bifidobacterium bifidum*, a group of probiotic bacterial strains, effectively alleviates LPS-induced autophagy and significantly ameliorates and restores physical barrier integrity that is diminished by LPS treatment in rat intestinal epithelial cells [15]. To our knowledge, reports on how the Lactobacillus species regulate the autophagy are rare.

Adhesive ability of probiotics to intestinal surfaces is believed to be of critical importance to confer benefit to the host. Two high adhesive strains of Lactobacillus exerted anti-inflammatory effects on Salmonella-infected intestinal epithelial cells (IEC) [16]. *L.* fermentum I5007, a high adhesion strain isolated from the gastrointestinal tract of piglet, showed beneficial effects on the gut epithelial barrier in neonatal piglets [17]. Our group isolated a novel *Lactobacillus* reuteri strain with high adhesive ability (adhesion indexes of 12.35 ± 0.09 CFU/cell), named ZJ617 [16], of which surface protein GAPDH serves as a key adhesion component [18]. This strain showed great tolerance to heat, acid, bile salt, Zn^2+^ and Cu^2+^, as well as antagonism to pathogenic agents [16]. Subsequently, our studies demonstrated that this strain could ameliorate inflammation and modulate the intestinal immune response and metabolism in LPS-induced mice [19]. Collectively, our present researches indicate that *Lactobacillus reuteri* ZJ617 can be used as a novel probiotic candidate. LGG is a widely studied probiotic that has well-documented adhesive properties. Therefore, LGG as a model of probiotic bacterium was compared with ZJ617 in light of their effects on gut integrity in this study. In the current experiments, we elaborated whether the probiotic ZJ617 could attenuate LPS-induced intestinal barrier dysfunction through its anti-inflammation, antioxidant and regulating effect on the tight junction, apoptosis and autophagy.

## RESULTS

### Oral administration of LAB attenuated the LPS-induced oxidative stress in the serum

**Figure 1:**
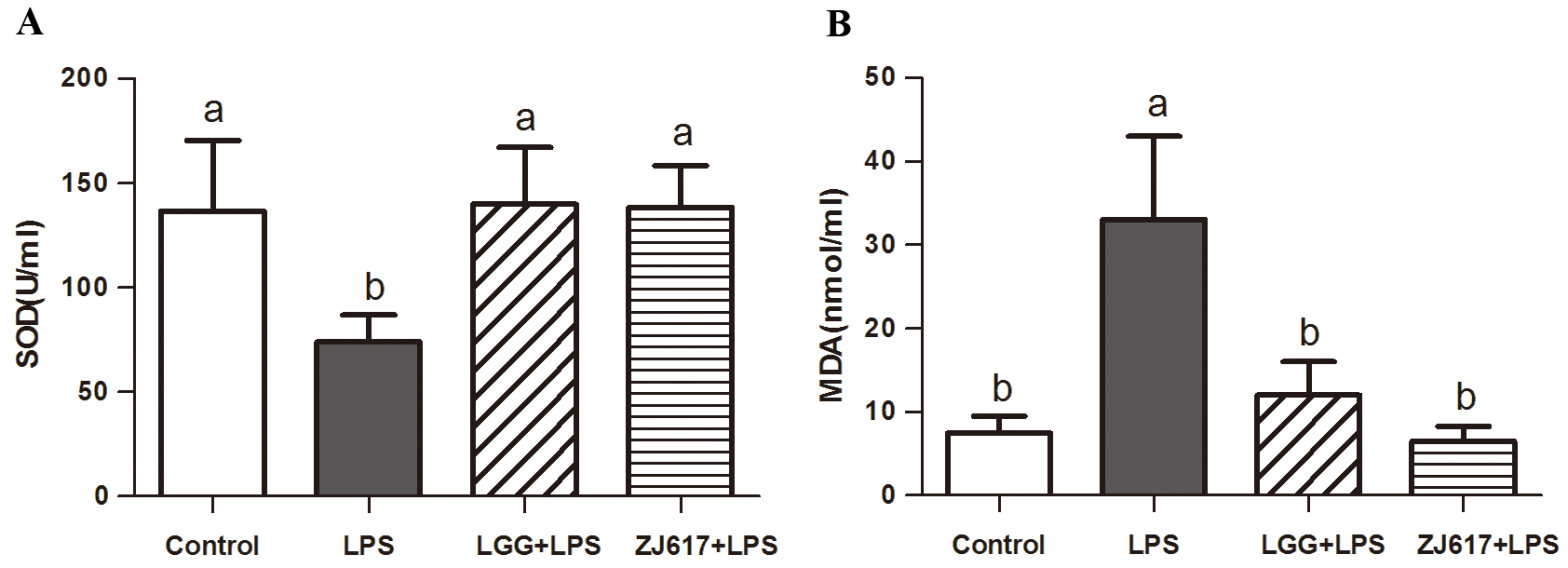
Effect of Lactobacillus rhamnosus GG (LGG) and Lactobacillus reuteri ZJ617 (ZJ617) on LPS-induced oxidative damage Mice were orally inoculated with lactobacilli for 7 days before the challenge with an intraperitoneal injection of 10 mg/kg LPS. Blood samples were obtained 24 h after injection to evaluate serum superoxide dismutase (SOD) activity **(A)** and malondialdehyde (MDA) levels **(B)**. The values are expressed as means ± SD (n = 6). Different alphabet indicates significance (*P* < 0.05).

Oxidative stress in the serum was assessed by measuring the activity of SOD (Figure 1A) and the level of MDA (Figure 1B). In our mice model the SOD activity in serum significantly decreased after the challenge with LPS (*P* < 0.05, Figure 1A). However, administration of the ZJ617 and LGG strain restored SOD levels (Figure 1A). LPS treatment caused an increase in the MDA levels, while LGG and ZJ617 supplementation decreased the levels of MDA that reached the normal values (Figure 1B).

### LAB treatment decreases LPS-induced intestinal permeability in mice

**Figure 2:**
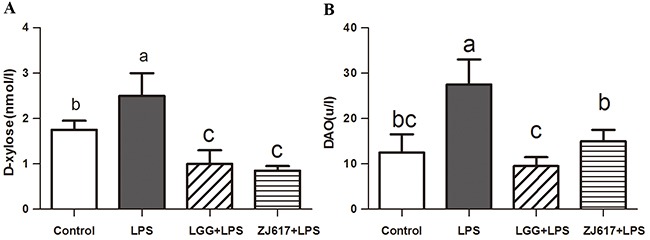
Effect of Lactobacillus rhamnosus GG (LGG) and Lactobacillus reuteri ZJ617 (ZJ617) on LPS-induced intestinal integrity Mice were orally inoculated with lactobacilli for 7 days before the challenge with an intraperitoneal injection of 10 mg/kg LPS. Blood samples were obtained 24 h after injection of LPS to evaluate serum D-xylose levels **(A)** and diamine oxidase activities (DAO) **(B)**. The values are expressed as means ± SD (n = 6). Different alphabet indicates significance (*P* < 0.05).

To investigate the probiotic effect of ZJ617 on intestinal permeability of mice challenged with LPS, serum D-xylose concentrations (Figure 2A) and DAO activity (Figure 2B) were measured as markers of intestine integrity. We observed that challenge with LPS significantly increased serum D-xylose (*P* < 0.05, Figure 2A) and DAO (*P* < 0.05, Figure 2B) and treatment with LGG and ZJ617 significantly reduced them.

### LAB supplementation attenuates LPS-induced tight junction protein expression in the ileum

**Figure 3:**
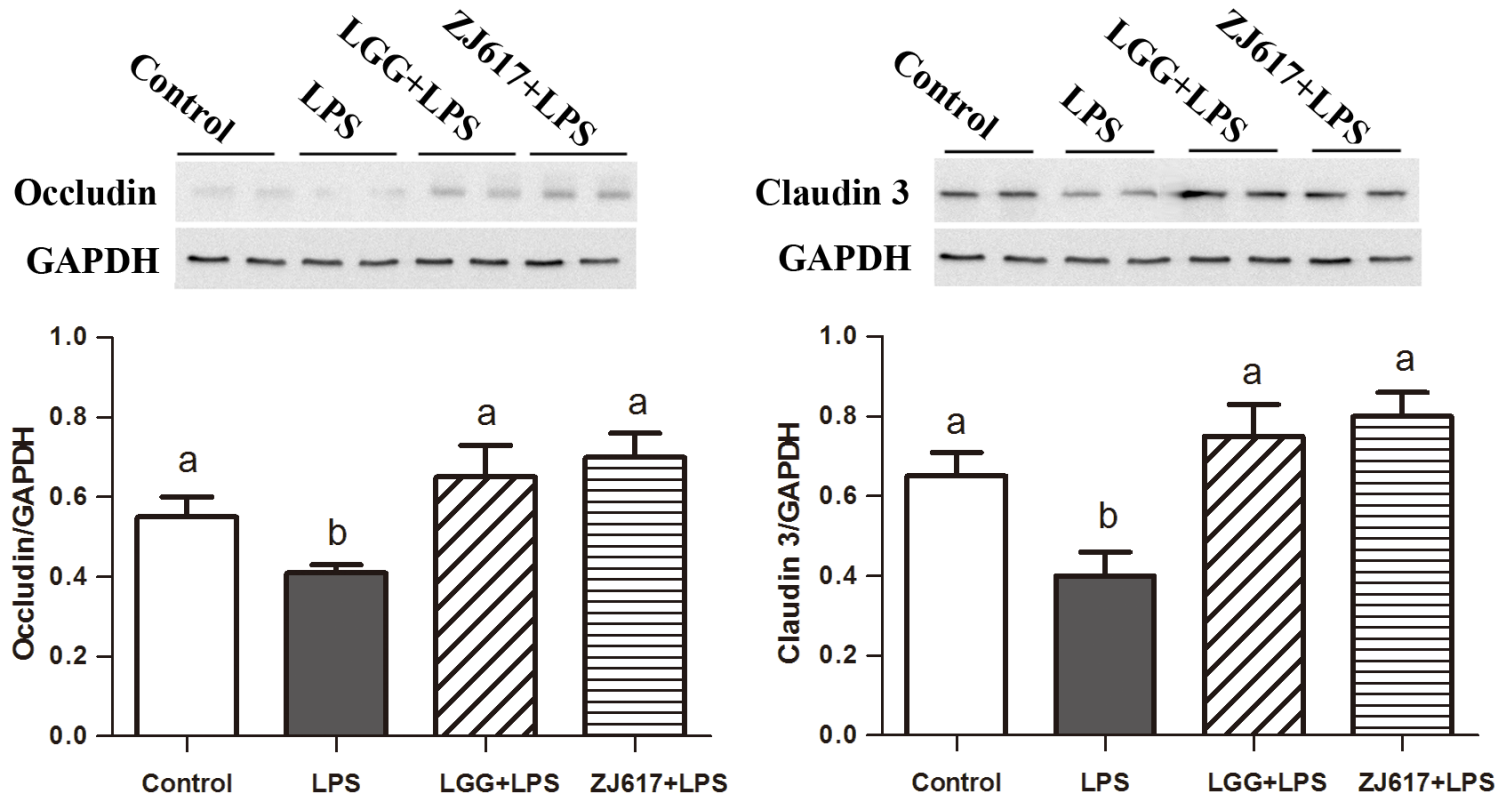
Effect of Lactobacillus rhamnosus GG (LGG) and Lactobacillus reuteri ZJ617 (ZJ617) on intestinal tight junctions in mice challenged with LPS Mice were orally inoculated with lactobacilli for 7 days before the challenge with an intraperitoneal injection of 10 mg/kg LPS. Ileal tissues from six mice in each group were obtained 24 h after injection. Immunoblotting of and quantitative analysis of ileal occludin and claudin 3. The protein bands were quantified by densitometry analysis, normalized to GAPDH. The values are expressed as means ± SD (n = 6). Different alphabet indicates significance (*P* < 0.05).

**Figure 4:**
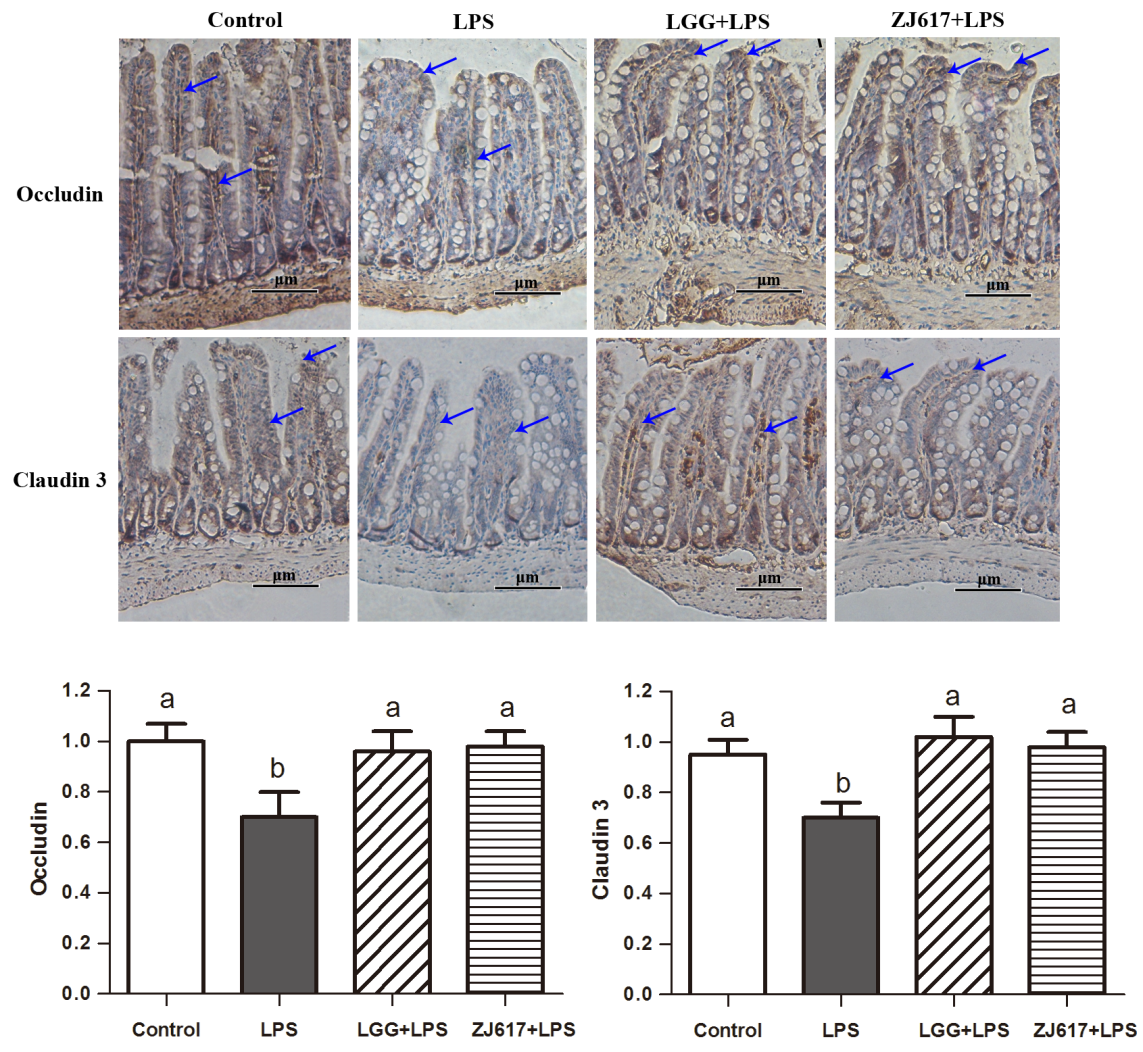
Effect of Lactobacillus rhamnosus GG (LGG) and Lactobacillus reuteri ZJ617 (ZJ617) on intestinal tight junctions in mice challenged with LPS Mice were orally inoculated with lactobacilli for 7 days before the challenge with an intraperitoneal injection of 10 mg/kg LPS. Ileal tissues from six mice in each group were obtained 24 h after injection. Immunohistochemistry of and quantitative analysis of ileal occludin and claudin 3. Brown staining indicates occludin and claudin 3 positive cells. The ratio of positive-stained cells/all cells is expressed as the mean ± SD (n= 6). Different alphabet indicates significance (*P* < 0.05).

Occludin and claudin-3, tight junction proteins, play a vital role in gut permeability. Western blotting analysis reveled that a significant decrease in the both proteins abundance after LPS stimulation; LGG and ZJ617 treatment attenuated this reduction (*P* < 0.05, Figure 3). Consistent with the results of western-blot analysis, IHC analysis indicated that LPS stimulation caused a reduction in abundance of both occludin and claudin-3, and LGG and ZJ617 treatment normalized tight junction protein expression (*P* < 0.05, Figure 4).

### LAB treatment ameliorates LPS-induced apoptosis in the ileum

**Figure 5:**
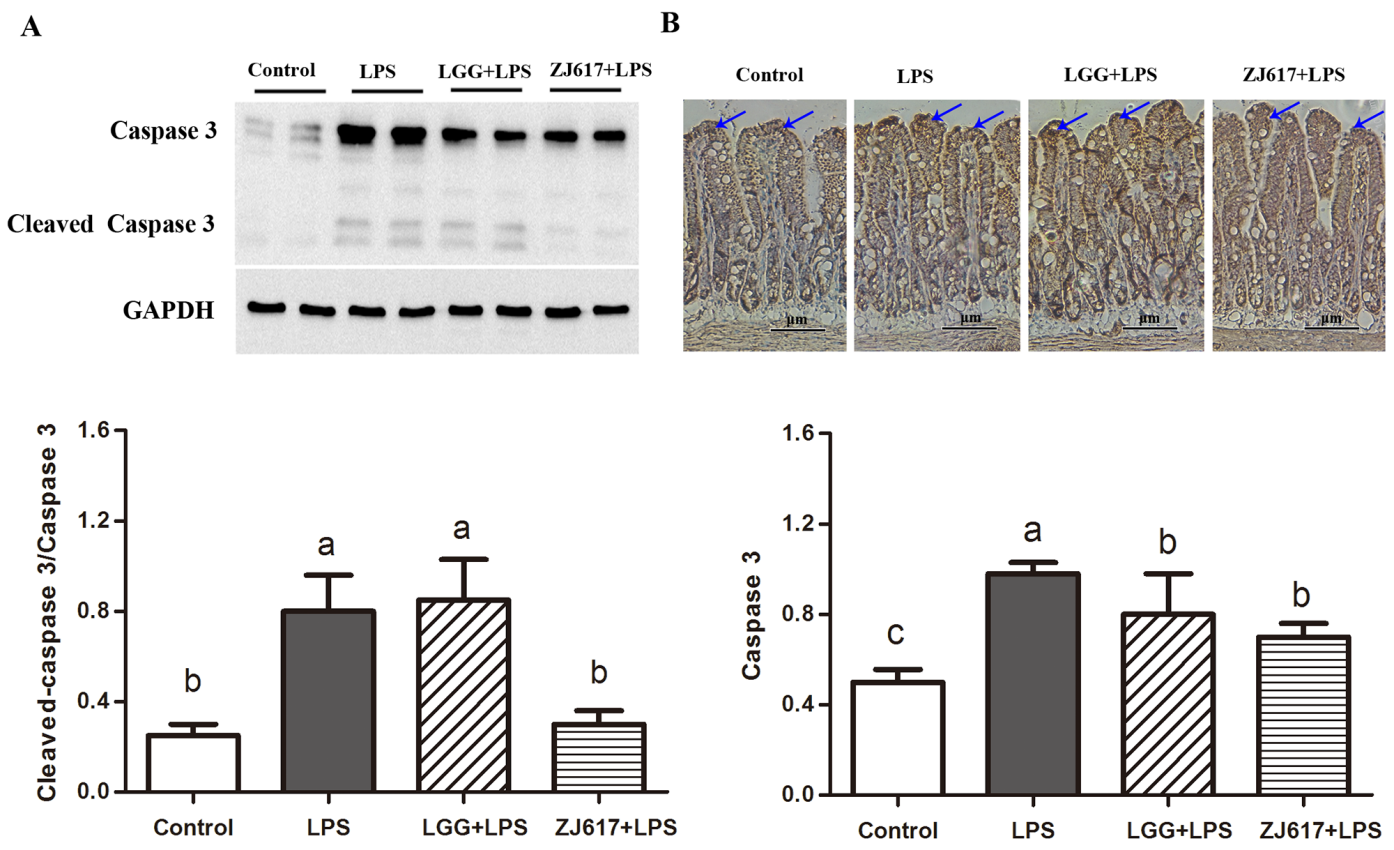
Effect of Lactobacillus rhamnosus GG (LGG) and Lactobacillus reuteri ZJ617 (ZJ617) on intestinal apoptosis in mice challenged with LPS Mice were orally inoculated with lactobacilli for 7 days before the challenge with an intraperitoneal injection of 10 mg/kg LPS. Ileal tissues from six mice in each group were obtained 24 h after injection. **(A)** Immunoblotting of and quantitative analysis of ileal caspase 3. The protein bands were quantified by densitometry analysis, normalized to GAPDH. The values are expressed as means ± SD (n = 6). Different alphabet indicates significance (*P* < 0.05). **(B)** Immunohistochemistry of and quantitative analysis of ileal caspase 3. Brown staining indicates caspase 3 positive cells. The ratio of positive-stained cells/all cells is expressed as the mean ± SD (n= 6). Different alphabet indicates significance (*P* < 0.05).

To evaluate the effects of probiotic LAB on apoptosis of mice challenged with LPS, we investigated an apoptosis marker caspase-3. LPS treatment contributed to up-regulation of caspase-3, while ZJ617 rather than LGG decreased its expression to the normal level (Figure 5A). In line with the results of western-blot analysis, IHC analysis indicated that LPS stimulation increased caspase-3 activity, and such increase was attenuated by LGG and ZJ617 treatment (*P* < 0.05, Figure 5B).

### LAB treatment protects the intestine from LPS-induced autophagy in the ileum

**Figure 6:**
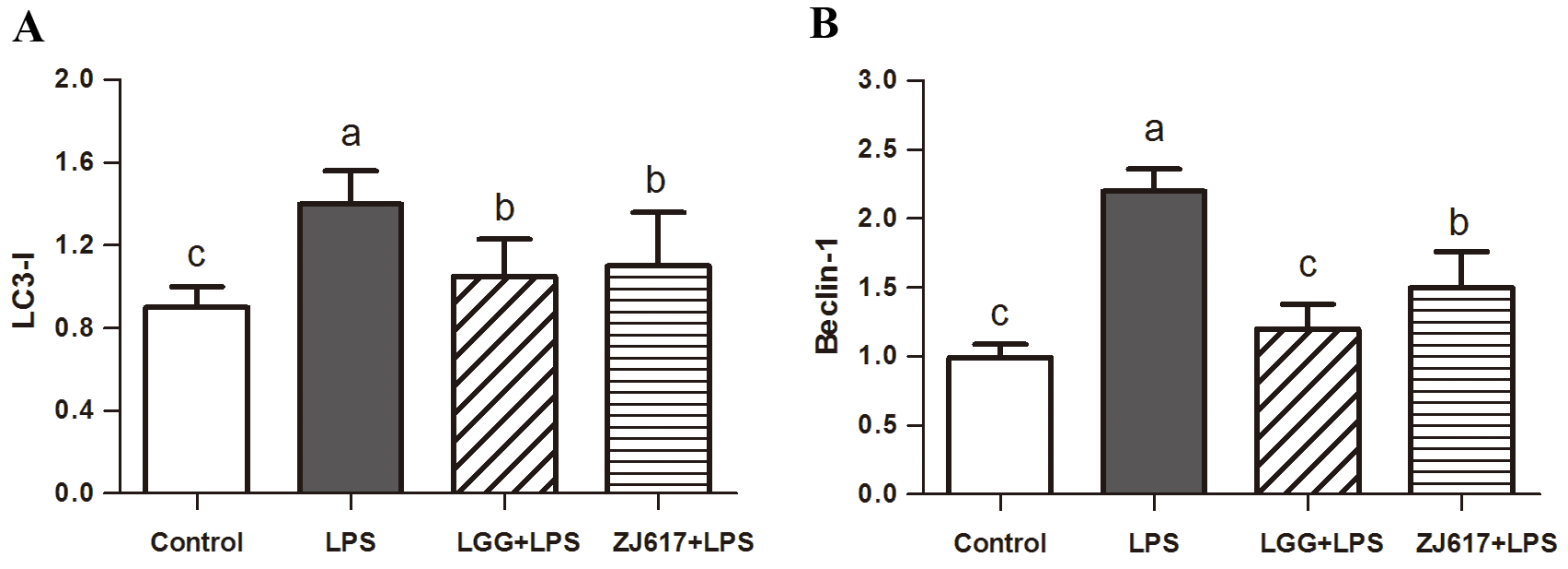
Effect of Lactobacillus rhamnosus GG (LGG) and Lactobacillus reuteri ZJ617 (ZJ617) on intestinal autophagy in mice challenged with LPS Mice were orally inoculated with lactobacilli for 7 days before the challenge with an intraperitoneal injection of 10 mg/kg LPS. Ileal tissues from six mice in each group were obtained 24 h after injection. mRNA levels of microtubule-associated light chain 3 (LC 3) **(A)** and beclin-1 **(B)** were examined by qRT-PCR. The values are expressed as means ± SD (n = 6). Different alphabet indicates significance (*P* < 0.05).

**Figure 7:**
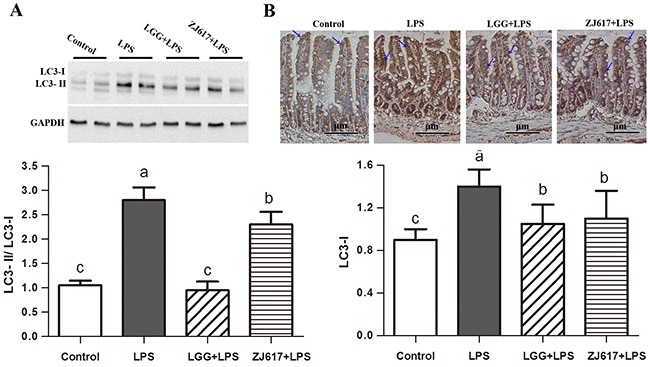
Effect of Lactobacillus rhamnosus GG (LGG) and Lactobacillus reuteri ZJ617 (ZJ617) on intestinal autophagy in mice challenged with LPS Mice were orally inoculated with lactobacilli for 7 days before the challenge with an intraperitoneal injection of 10 mg/kg LPS. Ileal tissues from six mice in each group were obtained 24 h after injection. **(A)** Immunoblotting of and quantitative analysis of ileal microtubule-associated light chain 3 (LC3). The protein bands were quantified by densitometry analysis, normalized to GAPDH. The values are expressed as means ± SD (n = 6). **(B)** Immunohistochemistry of and quantitative analysis of ileal LC3. Brown staining indicates LC3 positive cells. The ratio of positive-stained cells/all cells is expressed as the mean ± SD (n= 6). Different alphabet indicates significance (*P* < 0.05).

**Figure 8:**
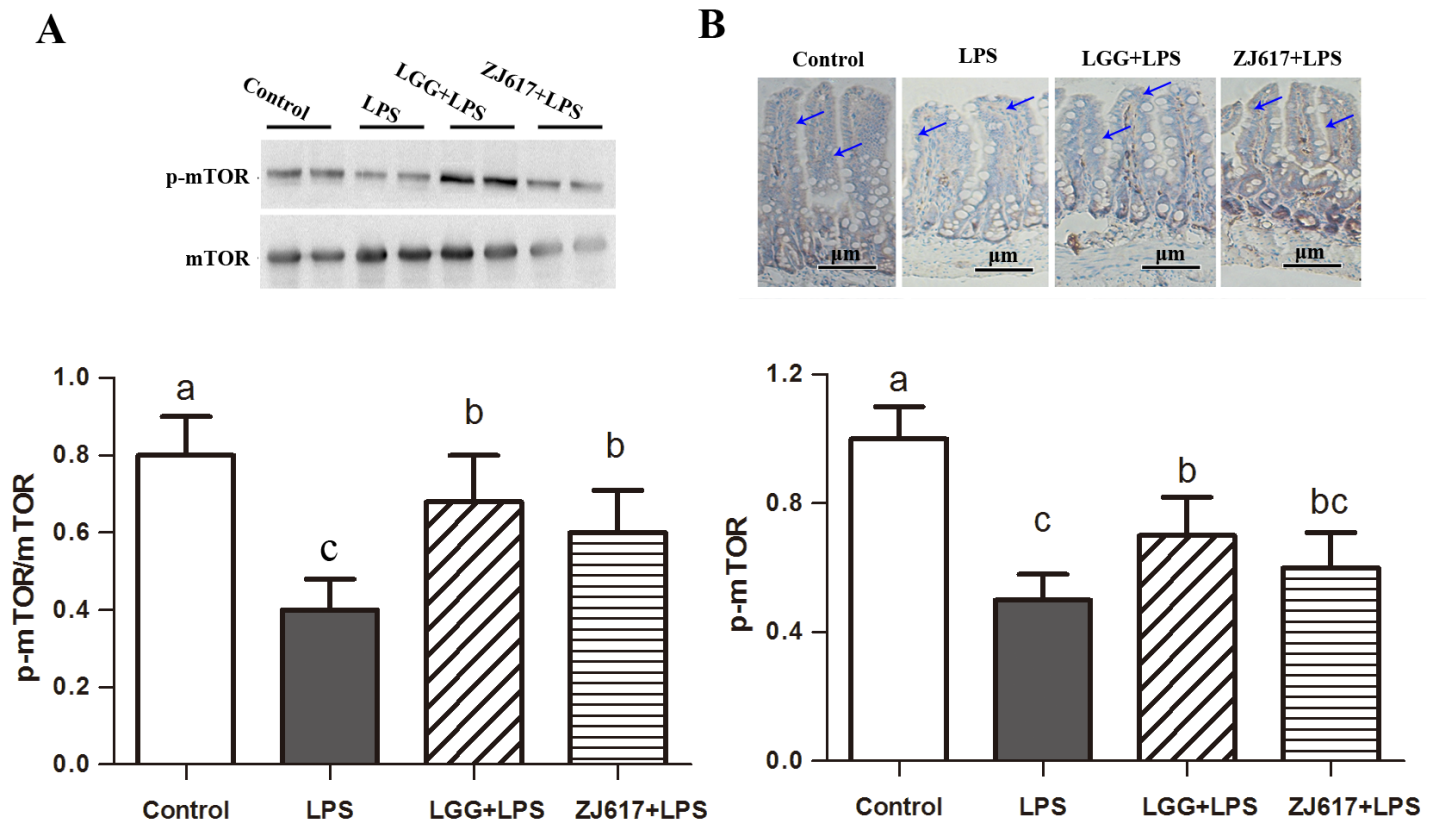
Effect of Lactobacillus rhamnosus GG (LGG) and Lactobacillus reuteri ZJ617 (ZJ617) on intestinal autophagy in mice challenged with LPS Mice were orally inoculated with lactobacilli for 7 days before the challenge with an intraperitoneal injection of 10 mg/kg LPS. Ileal tissues from six mice in each group were obtained 24 h after injection. Immunoblotting of and quantitative analysis of ileal mammalian target of rapamycin (mTOR). **(A)** Immunoblotting of and quantitative analysis of ileal mTOR. The protein bands were quantified by densitometry analysis, normalized to GAPDH. The values are expressed as means ± SD (n = 6). **(B)** Immunohistochemistry of and quantitative analysis of ileal mTOR. Brown staining indicates mTOR positive cells. The ratio of positive-stained cells/all cells is expressed as the mean ± SD (n= 6). Different alphabet indicates significance (*P* < 0.05).

To determine the effects of probiotic LAB on autophagy of mice challenged with LPS, we investigated autophagy markers beclin-1 and LC3-II and an autophagy initial regulator mTOR in the ileum (Figures 6-8). Challenge with LPS significantly (*P* < 0.05) increased the beclin-1 and LC3-II mRNA expression by qRT-PCR; ZJ617 and LGG supplementation inhibited such increase (*P* < 0.05, Figure 6). These findings were confirmed by western blot and IHC analysis for LC3, noting that administration of the ZJ617 and LGG strain significantly ameliorated LPS-induced overexpression of LC3-II (*P* < 0.05, Figure 7). LPS-induced downregulation of p-mTOR was observed by western blot, while ZJ617 and LGG increased its expression (*P* < 0.05, Figure 8).

## DISCUSSION

Our previous experiment [19] demonstrated that LGG, a reference strain, and ZJ617, with high adhesive ability, significantly reduced inflammatory damage during endotoxic shock induced by LPS administration in mice. Many studies have shown that oxidative stress and inflammation can interact in many conditions. For example, reactive oxygen species (ROS) scavengers can not only increase activities of antioxidant enzymes (superoxide dismutase, catalase, and glutathione peroxidase), but also improve the levels of IL-10 and decrease TNF-α and other pro-inflammatory cytokines [20]. On the other hand, a growing number of studies have demonstrated that several probiotics LAB acting as anti-inflammation are able to scavenge ROS by improving SOD activities and GSH levels [21, 22]. In support of these reports, our findings suggest that ZJ617 and LGG could strongly reverse the damage associated with the LPS-induced redox imbalance.

Intestinal epithelial barrier is primarily composed of epithelial cells and the tight junctions (TJ) between them [23]. Disruption of tight junctions can increase the gut permeability. Occludin and claudins are two main types of transcellular proteins in TJ proteins. Our previous and present studies show that challenge with LPS increases the formation of cytokines and ROS in mice, both of which can disrupt the TJ and compromise the barrier function of the intestinal epithelium [11, 24]. Many studies have shown that pretreatment with probiotic bacteria can inhibit the decrease in resistance and TJ alteration caused by stress or proinflammatory cytokines [25, 26]. Compared with LPS group, pretreatment with LGG and ZJ617 significantly reduced intestine permeability, and increased expression of occludin and claudin 3. These data support the notion that ZJ617 and LGG pretreatments contribute to a decrease in oxidative stress and inflammation, which in turn, lead to improved tight junction protein expression and thus to restoration of barrier function.

On the other hand, epithelial barrier permeability is attributed to regulation of TJ structure which is achieved via myosin light chain II (MLC) phosphorylation [27]. MLC kinase (MLCK), MAP kinases ERK1/2 and p38 can all phosphorylate MLC causing increased permeability [28]. Our previous studies demonstrated that ZJ617 and LGG could significantly suppress LPS-induced phosphorylation of p38 MAPK, ERK1/2, which may lead to decrease in MLC phosphorylation. Taken together, these results indicate that ZJ617 and LGG protect the intestinal epithelial tight junctions and the barrier function from LPS-induced insult by restoration of TJ protein expression and maintenance of TJ structure via MAP kinase-dependent mechanism.

Epithelial turnover, including cell proliferation and apoptosis, play a critical role in maintenance of intestinal integrity [29, 30]. Epithelial cell apoptosis increase has been considered to be the main mechanism underling the intestinal mucosal injury. Increased permeability of the epithelial barrier can be caused by apoptosis via caspase-3 activation [31]. In the current study, ZJ617 supplementation reduced apoptosis induced by LPS challenge. In support of our results, previous reports demonstrated that the probiotic bacterium *Lactobacillus rhamnosus* prevented cytokine-induced apoptosis in epithelial cell lines [14]. These results suggest that ZJ617 supplementation may protect intestinal integrity by inhibiting epithelial cell apoptosis, although the mechanism of the *Lactobacillus* regulation of cell apoptosis has not been fully elucidated. A previous study has reported that LGG prevents cytokine-induced apoptosis in both human and mouse intestinal epithelial cells through inhibiting p38 MAPK activation [32].

Autophagy is regarded as an essential cell survival mechanism involved in the intestinal barrier function. However, excessive autophagy can bring about pathological conditions and finally to autophagic cell death [33]. LPS has acted as stimulator of autophagic signaling in cultured intestinal epithelial cells and RAW 264.7 cells [15, 34]. A recent study has shown that probiotic *bifidobacterium bifidum* treatment could effectively inhibit LPS-induced autophagy by preventing autophagolysosomal fusion in rat IEC18 cells [15]. The LC3 is now widely used to monitor autophagy and acts as a good early marker for the formation of autophagosomes. mTOR signaling is a well-known pathway for autophagy induction. Interestingly, we found that p-mTOR expression was reduced but LC3-II expression was elevated by LPS challenge; however, such effect was notably attenuated by LGG and ZJ617. These results are consistent with previous studies showing that mTOR is the major negative regulator of autophagy [35, 36]. Similarly, LGG suppresses human rotavirus-induced autophagy in the gnotobiotic piglet intestine [37]. Our results suggest that treatment with probiotics may suppress increased autophagy via mTOR-dependent pathway and thus protect against intestinal epithelial barrier disruption induced by LPS.

## CONCLUSION

*Lactobacillus reuteri* ZJ617 with high adhesive ability could improve intestinal integrity during endotoxic shock. Its protective effects on the intestine are probably related to alleviating oxidative stress, improving tight junction protein expression, inhibiting apoptosis and autophaugy via mTOR-dependent pathway. The beneficial role of *Lactobacillus reuteri* ZJ617 may have strong potential for alleviating some inflammation taking place in intestine.

## MATERIALS AND METHODS

### Bacterial strains and culture

*Lactobacillus rhamnosus* GG (LGG) was a gift from Prof. Jinru Chen at the University of Georgia. *Lactobacillus reuteri* ZJ617 with high adhesive abilities were previously isolated from piglet intestine by our experiment colleagues [16]. LGG and ZJ617 were anaerobically grown at 37°C in MRS (de Man, Rogosa and Sharpe medium, Britania, Buenos Aires, Argentina) for 18h. The microorganisms viability was determined by colony forming units (CFU) that were counted by dilution and streaking on MRS agar plates (Difco) at 37°C overnight. Bacteria were harvested at the logarithmic growth phase by centrifugation at 4 °C (5 min, 4000 *g*), washed twice with phosphate-buffered saline (PBS) and finally suspended in this medium at a concentration of 1 × 10^8^ CFU/ml.

### Experiment treatment and sample collection

C57BL/6 mice (20 ± 2 g, 6-8 weeks old) were purchased from Model Animal Research Center of Nanjing University (Nanjing, China). Animal care was performed in accordance with guidelines of the Zhejiang Ethics Committee and received prior approval from the Animal Care and Use Committee in Zhejiang A & F University. Mice were kept at a constant temperature of 26 ± 2 °C with 12 h light–dark cycles and provided with *ad libitum* feed intake and water. The mice were randomly assigned to four groups (n= 6): two of them, designed as control and LPS groups, received PBS. The others, named as LGG + LPS and ZJ617 + LPS respectively, orally inoculated with LGG or ZJ617 suspended in sterile PBS at the concentration of 1×108 CFU/ml daily prepared respectively. At 7 days after starting the oral administration of the strain, LPS stimulation was conducted in mouse from LPS, LGG + LPS and ZJ617 + LPS groups with an intraperitoneal (i.p.) injection of 10mg/kg LPS respectively. The control groups received an i.p. injection of sterile PBS. The used does of LPS did not induce mice death in the following 24 h determined by our previous study [19]. Blood samples and ileum tissues (5 cm proximal from the ileal-cecal junction) were collected 24 h after the LPS challenge.

### Biochemical assays

Blood samples were collected by cardiac puncture. Serum samples were obtained by centrifuging the blood at 3000 g for 15 minutes at 4°C. Inflammatory cytokine TNF-α, D-xylose and malondialdehyde (MDA) concentrations in serum were assayed by ELISA kit (Nanjing Jiancheng Bioengineering Institution, Nanjing, China) according to manufacturer's instruction. Activities of superoxidase dismutase (SOD) and diamine oxidase (DAO) in serum were determined by a kinetic based assay using commercially available kits (Nanjing Jiancheng Bioengineering Institution, Nanjing, China).

### Real-time quantitative RT-PCR assay

The mRNA levels were determined by real-time quantitative RT-PCR (qRT-PCR). In brief, total RNA was prepared from each tissue sample (50 mg) using TRIzol reagent according to the manufacturer's protocol according to the manufacturer's instructions (Invitrogen Life Technologies, USA). cDNA synthesis was conducted using the HiFi-MMLV cDNA first strand synthesis kit (CWbio Co., Ltd, Cat#CW0744, China). The gene-specific primer pairs are listed in Table 1. qRT-PCR was performed using an ABI 7500 real-time PCR thermocycler instrument (ABI, Norwalk, CT). qRT-PCR was conducted in a 20-μl reaction system containing 1 μl cDNA, 0.5 μl forward primers (10 μM), 0.5 μl reverse primers (10 μM), 10 μl SYBR Green Supermix, and 8.0 μl water. The fold change was calculated using the 2^−ΔΔCt^ method [38].

**Table 1:** Primer sequences for the PCR amplification of specific genes

Gene	NCBI accession NO.	Primer sequence	Sense 5’– 3’	Primer size (bp)	Product size (bp)
			Antisense 5 ’– 3 ’		
***LC 3***	NM_026160.4	CCGAGAAGACCTTCAAGCAG		20	288
		ACACTTCGGAGATGGGAGTG		20	
***Beclin-1***	XM_006533784.3	TGATCCAGGAGCTGGAAGAT		20	299
		CAAGCGACCCAGTCTGAAAT		20	
***GAPDH***	XM_017321385.1	CCCCATAATAACAGGAGGGGC		21	134
		GCTTCACCTCCCCATACACAC		21	

### Western blot

Ileum tissues were lysed using lysis buffer (Sigma-Aldrich) in liquid nitrogen according to the manufacturer's instructions. The concentration of protein in samples was determined by Bradford's method [39]. Total protein (30μg/sample) was separated by electrophoresis (Bio-Rad, Richmond, CA, USA) on 10% SDS-PAGE, and transferred to a PVDF membrane (Millipore, Billerica, MA, USA). The blotted membrane was blocked for 2 h at room temperature in 1 × TBST [0.05% Tween 20, 100 mM Tris–HCl and 150 mM NaCl (pH 7.5)] containing 5% fat-free dry milk, and then incubated under gentle agitation all the night at room temperature in the presence of the primary antibodies: occludin, 1:5000 dilution of purified rabbit polyclonal anti-occludin antibody (Abcam, AB31721, Cambridge, UK); claudin-3, 1: 5000 dilution of purified rabbit polyclonal anti- claudin-3 antibody (Abcam, AB15102, Cambridge, UK); caspase-3, 1:5000 dilution of purified rabbit polyclonal anti- caspase-3 antibody (Abcam, AB13847, Cambridge, UK); glyceraldehyde-3-phophate dehydrogenase (GAPDH), 1:1000 dilution of purified rabbit monoclonal anti-GAPDH antibody (CST, D16H11, Danvers, USA); LC3, 1:1000 dilution of purified rabbit monoclonal anti-LC3 protein antibody (CST, D3U4C, Danvers, USA); mTOR, 1:1000 dilution of purified rabbit monoclonal anti-mTOR protein antibody (CST, 7C10, Danvers, USA); p-mTOR, 1:1000 dilution of purified rabbit monoclonal anti- p-mTOR protein antibody (CST, D3F9, Danvers, USA), which were able to bind to their specific protein. The blots were extensively washed with TBST buffer for 10 min × 3 times and incubated under gentle agitation with the secondary antibodies for immunodetection. The antigenantibody interaction was carried out for 1 h, and the crossreacting proteins were detected using ECL (Perkin Elmer Life Sciences, Boston, MA, USA). The protein bands were visualized with a chemiluminescence substrate using a gel-imaging system (Tanon Science and Technology, Shanghai, China) with Image Analysis Software (National Institutes of Health, Bethesda, MD, USA). In all instances, density values of bands were corrected by subtraction of the background values. GAPDH was used as the internal reference protein. Bands were standardized to the density of GAPDH and normalized fold expression represented as a ratio of each protein to GAPDH.

### Immunohistochemistry

Ileum tissues were fixed in formalin, embedded in paraffin, and cut into 4 μm sections according to previous methods [40]. Deparaffinized and rehydrated sections were blocked with 10 % normal goat serum for 1 h. Sections were incubated with the rabbit anti- occludin (1:5000), anti-claudin3 (1:5000), anti-caspase-3 (1:5000), anti-LC3 (1:1000) and anti-mTOR (1:1000) overnight at 4 °C, then with HRP-conjugated secondary antibody for 1 h. The Diaminobenzidine-HRP detection system was added, sections were then counterstained with hematoxylin, dehydrated and cover-slipped. Assessment of positivity of IHC staining [41] were conducted under a microscope (ECLIPSE Ti, Nikon Corp., Tokyo, Japan).

### Statistical analysis

Thee values of experimental data were presented as means ± standard deviation (SD) of the replicates. The statistical significance was analyzed using one-way analysis of variance (ANOVA), followed by Duncan's multiple range test using the SAS program (SAS Institute, INC, USA). Differences between groups are considered significant at *P* values < 0.05.
